# Drug Induced Sleep Endoscopy Identification of Adenoid Regrowth in Pediatric Obstructive Sleep Apnea

**DOI:** 10.1155/2018/7920907

**Published:** 2018-04-26

**Authors:** Habib G. Zalzal, Michele Carr, Nainika Nanda, Steven Coutras

**Affiliations:** ^1^Department of Otolaryngology, Head and Neck Surgery, West Virginia University School of Medicine, Morgantown, WV, USA; ^2^West Virginia University School of Medicine, Morgantown, WV, USA

## Abstract

**Objective:**

To establish the incidence and possible contributing factors leading to adenoid regrowth in children with pediatric sleep apnea using drug induced sleep endoscopy (DISE).

**Methods:**

Children treated for obstructive sleep apnea following previous adenoidectomy were evaluated using DISE. Adenoid regrowth was scored by the same attending physician using a 5-point grading scale. Age, sex, body mass index (BMI) percent for age, polysomnogram results, initial adenoid size before adenoidectomy, and postoperative complications were evaluated.

**Results:**

Fifty-six patients (age range 22 months to 16 years) met inclusion criteria. Twenty-five children (44.6%) had Grade 2 adenoid or larger. Mean age at the time of DISE was 7.11 years, with an average of 1.75 years since initial adenoidectomy. Mean preadenoidectomy size based on intraoperative nasopharyngeal mirror assessment was Grade 2.55 (95% CI 2.30–2.79). Adenoid size at time of sleep endoscopy was Grade 1.64 (95% CI 1.30–1.98). Characteristics associated with adenoid regrowth were higher body mass index for age percentile at time of endoscopy (*P* < 0.05), initial adenoid size (*P* < 0.01), and time between initial adenoidectomy and endoscopy (*P* = 0.05).

**Conclusions:**

Body mass index for age percentile, initial adenoid size, and time between initial adenoidectomy and drug induced sleep endoscopy correlate with regrowth in childhood obstructive sleep apnea.

## 1. Introduction

Adenoidectomy, with or without tonsillectomy, is one of the more frequently performed operations within otolaryngology, estimated to be about 129,540 operations per year in the United States [1, 2]. Indications for adenoidectomy range from adenoid-induced obstruction of the nasopharynx to chronic infections of the middle ear, sinuses, and adenoid itself. Often, symptoms resolve following successful surgical intervention, but recurrence of symptoms with adenoid regrowth has been documented in 1–25% of patients who have an adenoidectomy [2–4].

Obstructive sleep apnea (OSA) affects 1–4% of children [5]. In children who require revision adenoidectomy, up to 94% of patients have symptoms associated with nasopharyngeal obstruction including snoring and sleep disturbance [6]. Children with persistent OSA following adenotonsillectomy may undergo drug induced sleep endoscopy (DISE) to identify the location of upper airway obstruction. In a sedated patient, DISE allows for identification of sites of obstruction of the upper respiratory tract during simulated sleep and complete visualization of the upper respiratory tract. DISE is an effective tool for surgical planning in children with persistent OSA after adenotonsillectomy or if their physical exam is not consistent with adenotonsillar hypertrophy [7].

This study uses DISE as the main diagnostic tool to determine the incidence of adenoid regrowth at our institution and identify risk factors associated with recurrent symptoms leading to revision adenoidectomy.

## 2. Materials and Methods

**Figure 1:**
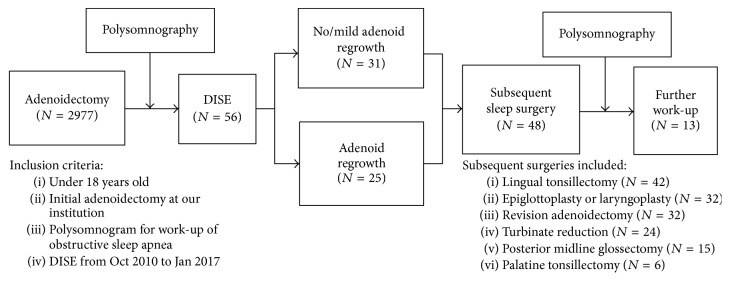
Methodology for inclusion criteria and data accumulation for obstructive sleep apnea patients undergoing drug induced sleep endoscopy (DISE).

This study was a retrospective case series with chart review performed at a large tertiary academic facility following institutional review board approval. A computational search using the EPIC electronic medical record was performed for the term “flexible laryngoscopy for sleep endoscopy,” consistent with the phrase used for DISE at our institution. Search was limited to patients under 18 years identified as having undergone polysomnography (PSG) prior to sleep endoscopy from October 13, 2010, to January 1, 2017 (Figure 1). All patients were managed by the same attending otolaryngologist. Sleep endoscopy was performed after a positive polysomnogram for sleep apnea with no obvious source of airway obstruction on physical exam.

Inclusion criteria involved children (age < 18 years) who underwent primary adenoidectomy with or without tonsillectomy at our institution and subsequently required DISE for evaluation of PSG confirmed persistent obstructive sleep apnea. Patients were excluded if they previously had primary adenoidectomy at an outside facility or did not have documentation of adenoid size during primary adenoidectomy. Data extracted from the electronic medical record included gender, birthdate, initial adenoidectomy technique, adenoid size per surgical and endoscopic evaluations, date of adenoidectomy, date of sleep endoscopy, body mass index (BMI) and weight at the time of sleep endoscopy, allergic rhinitis/allergies, asthma, behavior disorders, developmental delay, reflux, other comorbidities, and subsequent sleep surgeries. Six different otolaryngologists with resident assistance were responsible for the initial adenoidectomy of the patients since electronic medical record keeping began at this institution in 2009. Adenoidectomies were performed for a variety of reasons, the majority for OSA, but also for middle ear disease and chronic adenoiditis. Techniques employed included suction electrocautery or coblation of the adenoid tissue.

**Figure 2:**
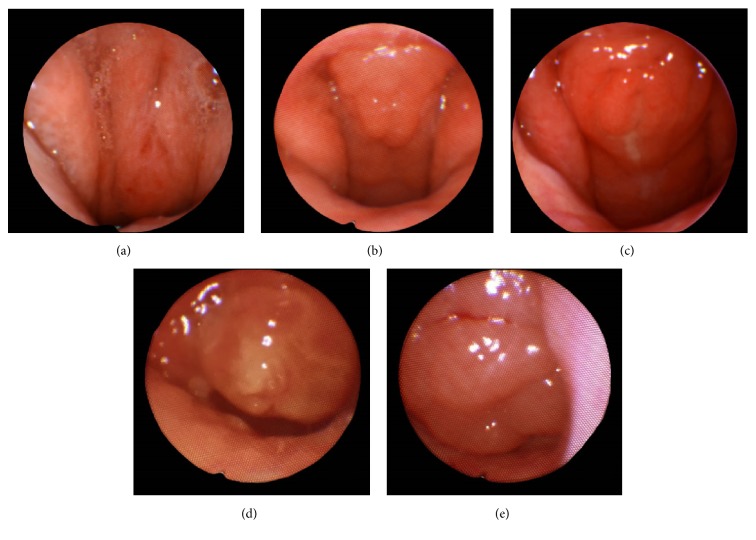
Adenoid hypertrophy grading system used at West Virginia University for Drug Induced Sleep Endoscopy. Grade 0 (0%) (a), Grade 1 (<40%) (b), Grade 2 (41%–70%) (c), Grade 3 (71%–90%) (d), and Grade 4 (91%–100%) (e).

Diagnosis of medical conditions (allergies, asthma, etc.) was based on their presence either in the medical notation by the treating otolaryngologist or within the medical record via* International Classification of Diseases, Tenth Revision *(ICD-10) codification at the time of initial sleep endoscopy. Adenoid size at the time of primary adenoidectomy (preoperative adenoid size) was obtained from the operative notes. Adenoid size at DISE was subjectively graded by the same attending surgeon using a descriptive scale used at our institution and reported in the operative note (Figure 2). An adenoid size of Grade 0 signifies 0% obstruction of the choanae with likely presence of scarring, Grade 1 signifies <40% obstruction, Grade 2 signifies 41–70% obstruction, Grade 3 signifies 71–90% obstruction, and Grade 4 signifies complete obstruction (91–100%) of the choanae with lymphoid tissue touching the soft palate at rest.

BMI for age percentile was separated into four groups: “underweight” if BMI was less than the 25th percentile, “normal weight” if between 25 and 84.9%, “overweight” if between 85 and 94.9%, and “obese” if in the 95th percentile or greater. Adenoid regrowth was present if endoscopic visualization of the adenoid showed a Grade 2 or larger adenoid on postadenoidectomy DISE. All children undergoing revision adenoidectomy had an adenoid of Grade 2 or larger.

Statistical evaluation was executed using* IBM*®* SPSS Statistics*® (Version 24.0. Armonk, NY: IBM Corp) software. Chi-square analysis was performed for nominal data with Pearson's correlation, while independent samples t-tests were performed for interval data (time between procedures and age characteristics). Ordinal data (BMI, adenoid size) was analyzed using Kruskal Wallis testing and Spearman's Rho correlation. Statistical significance was defined as *P* ≤ 0.05.

## 3. Results

Fifty-six patients (age range 22 months to 16 years) met the inclusion criteria for having undergone primary adenoidectomy prior to DISE.  Table 1 summarizes the demographic data of these patients. Mean age at the time of DISE was 7.11 years, with an average of 1.75 years since initial adenoidectomy. Many children in our study had a BMI greater than the 95th percentile for age (46.4%). Allergic rhinitis was common in this population (46.4%). For behavioral issues, the majority of diagnoses were attention deficit disorder (ADHD) and oppositional defiant disorder (ODD). Subsequent sleep surgery was performed after DISE in 86% of patients (Figure 1).

**Figure 3:**
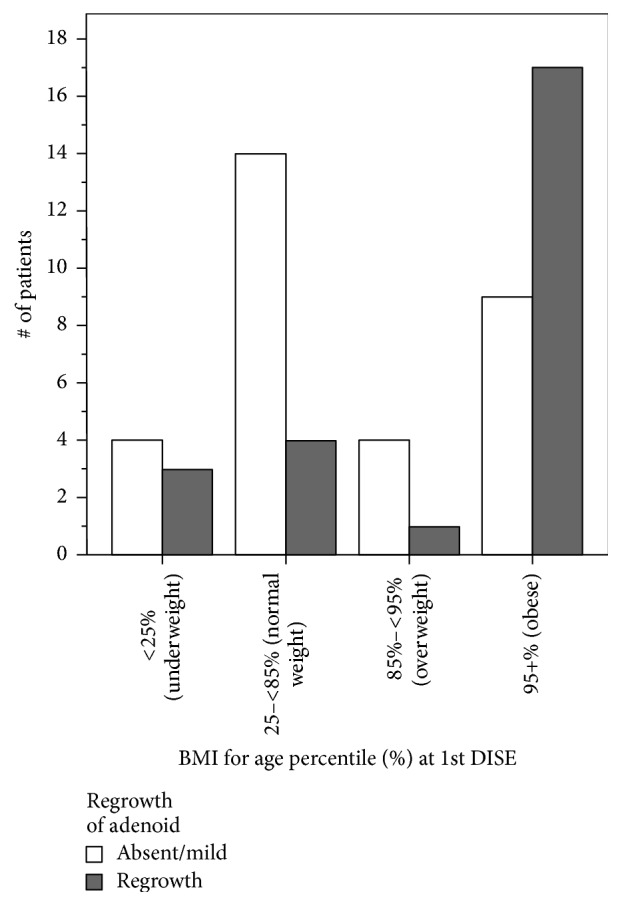
Relationship of body mass index (BMI) for age percentile with incidence of adenoid regrowth (*P* < 0.05).

**Figure 4:**
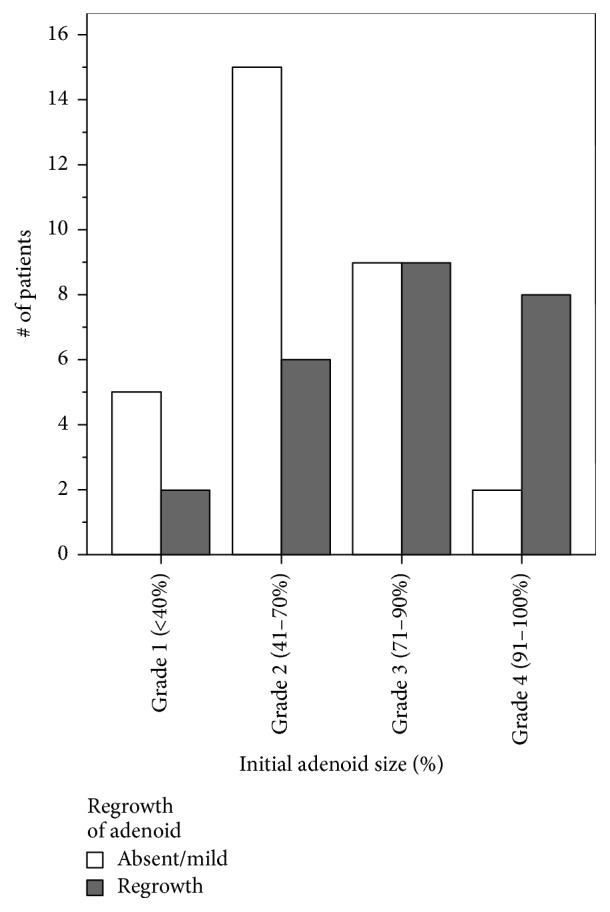
Relationship between initial adenoid size with subsequent adenoid regrowth (*P* < 0.01).

Adenoid characteristics are described in Table 2. The mean initial adenoid size based on preoperative assessment was Grade 2.55 (95% CI 2.30–2.79). Upon postoperative DISE, this improved to Grade 1.64 (95% CI 1.30–1.98). The incidence of regrowth as determined by DISE was 44.6%, and subsequently 32 patients underwent revision adenoidectomy. Factors associated with adenoid regrowth are summarized in Table 3: greater BMI percentile at the time of DISE (Kruskal Wallis *χ*^2^ = 5.559, *P* < 0.05; Figure 3), larger initial adenoid size (Kruskal Wallis *χ*^2^ = 7.013, *P* < 0.01; Figure 4), and increased time between initial adenoidectomy and DISE (*F* Statistic = 3.90, *P* = 0.05; 95% CI −1.70 to −0.06).

Adenoid regrowth had no significant correlation with other comorbidities, surgical technique, and initial surgery. Demographic data, age, and prior surgeries to adenoid size at first DISE were also not significant.

## 4. Discussion

Studies have shown that up to 94% of patients undergoing adenoid revision had problems with snoring, congestion, and noisy breathing prior to surgery [6]. However, other studies have demonstrated that most patients with adenoid regrowth up to about 40% obstruction are asymptomatic [3, 8]. Therefore, opinions exist that the recurrent obstructive symptoms in children with adenoid regrowth are due to underlying risk factors, such as age, body mass index, gender, and reflux, rather than the adenoid [9].

In our patient population of children with persistent OSA, 57% of patients underwent revision adenoidectomy. These patients presented with persistent symptoms and therefore represent a population more likely to have upper airway obstruction. This group has a higher rate of adenoid revision compared to other studies in the literature which focus on revision rate alone. A 2012 retrospective review of patients with mostly obstructive adenoids found the revision rate to be 2.2% of total pediatric adenoidectomies at 5 years and 3% at 10 years, with a higher risk of requiring revision in patients who had their first surgery at an earlier age [9]. However, our population was at risk for higher adenoid regrowth rates, since all subjects presented with persistent OSA. Similarly, a prospective analysis of 68 children with recurrent adenotonsillitis showed correlation between age and incidence of regrowth via transnasal endoscopy, with the most regrowth occurring in the group under age of 5 years [4]. Our study found no such relation between age and rate of regrowth as determined by DISE (Table 3, *P* = 0.31). Other studies have also demonstrated larger adenoid hypertrophy in younger children, especially those under age 5 years, and these children were most likely to undergo revision adenoidectomy [9, 10]. Age was an insignificant factor within our population most likely because DISE occurred after this natural growth period (7.11 years) [9, 10].

Significance was obtained when comparing elapsed time between initial adenoidectomy and first evaluation by DISE (Table 3, *P* = 0.05). Patients who had endoscopic visualization of their adenoid tissue soon after surgery would be unlikely to have regrowth compared to those with DISE in a later timeframe. Previous studies have shown adenoid size to increase for up to the first 12 years of life, consistent with this finding [2].

Our study found a relationship between adenoid size at initial surgery and adenoid regrowth (Table 3, Figure 4, *P* < 0.01). There was more obstruction at the time of initial surgery (40% with a Grade 2 adenoid) compared to obstruction at DISE (16.1% with Grade 2 adenoid). This is consistent with data published by Kim et al., who used cephalometric studies to determine that preoperative adenoid size was a predictor of regrowth [10]. Pediatric patients with Grade 2 or larger adenoids are more likely to experience regrowth compared to those who had a Grade 1 adenoid at the time of initial surgery, likely due to greater potential of adenoid growth in these children compared to patients with inherently smaller adenoid at first evaluation [10].

While some of these findings are consistent with what is known in the literature, specifically for technique and allergic rhinitis [9–13], other factors are not. An 11-year retrospective review of adenoidectomies found esophageal reflux disease in 40% of those undergoing revision surgery [5]. Children undergoing adenoidectomy alone were noted to be four times more likely to need revision compared to those who underwent an adenotonsillectomy [14]. Neither of these factors was significant in our study. In addition, while both ADHD and ODD have been associated with pediatric OSA, behavioral issues were not significant for adenoid regrowth either (Table 3, *P* = 0.083) [15].

Age percentile of BMI at time of first DISE was a notable predictor for regrowth in our patient population (Figure 3, *P* < 0.05). By contrast, other studies have noted no significant association between BMI and adenoid regrowth [10]. It is important to note that West Virginia has a high prevalence of obesity compared to other parts of the United States [16]. Nearly 50% of our study population was in the 95th percentile for BMI by age. Although no study to date has determined the relationship between BMI and adenoid regrowth, nitric oxide has previously been theorized to play a role in obese children with sleep apnea. Nitric oxide serves as a marker of airway inflammation [17]. Levels of expired nitric oxide are found at higher levels in children with obesity and sleep apnea, but not when compared to normal weight children with sleep apnea or obese children without sleep apnea [17, 18]. While adenoid size itself does not correlate with elevated nitric oxide levels [19], further work is needed to determine the association of adenoid regrowth to obese children with sleep apnea.

Within our population of persistently symptomatic patients, 44.6% of patients exhibited adenoid regrowth. Interestingly, most of these patients required a lingual tonsillectomy (*N* = 42; 75%) or laryngoplasty (*N* = 32, 57%) to resolve their airway obstruction, in addition to revision adenoidectomy (*N* = 32, 57%) at the time of other procedures. Overall 85.7% of patients underwent further surgery after sleep endoscopy. Other studies have demonstrated that adenotonsillectomy is often not curative for OSA, particularly in children who are older than 7 years or obese [20]. These findings are consistent with our study, confirming the value of DISE to locate other sources of obstruction in order to plan for further surgical intervention to reduce AHI in children with persistent disease [21]. Adenoid regrowth is unlikely to be the sole site of their airway obstruction during sleep.

A significant factor that could not be controlled in our study was surgical technique used for primary adenoidectomy, which may affect the incidence and degree of regrowth [11]. Since initial adenoidectomy was performed by six different otolaryngologists, surgical technique differed widely. Whether all adenoid tissue was removed could not be ascertained from the operative reports. Previous studies have shown that rate of adenoid regrowth is greatly reduced with adenoid removal down to the pharyngobasilar surface [13].

Our study had a relatively small sample size since we only included children with OSA who underwent DISE. This limits the power of the study to detect small differences.

Our group uses an adenoid size grading approach similar to that used in a 2011 study comparing endoscopic versus soft tissue lateral X-ray adenoid size. They used a 4-category endoscopic adenoid size grading scale based on percent of choanal obstruction [22]. We questioned whether adenoid size as judged by mirror exam was similar to size judged by nasal endoscopy. A study looking at 28 patients undergoing adenoidectomy found consistent correlation (*P* < 0.01) between adenoid size determined by endoscopy versus nasopharyngeal mirror examination [23]. Nevertheless, while our methods are not identical to other grading scales in the literature, endoscopic visualization of the adenoid is a useful tool for evaluating adenoid size in comparison with nasopharyngeal mirror exam [7].

## 5. Conclusions

Adenoidectomy remains a valuable procedure for children with sleep apnea. Our study found that initial adenoid size, BMI for age percentile, and time between adenoidectomy and DISE are the only significant predictors of subsequent adenoid regrowth in childhood obstructive sleep apnea. However, adenoid regrowth was not a major contributor to airway obstruction in 55.4% of children with obstructive sleep apnea, as over half of our patients needed surgery upon the hypopharyngeal or laryngeal airway as well in order to improve outcomes. This fact argues against revision adenoidectomy without sleep endoscopy in children with persistent OSA after adenotonsillectomy.

## Figures and Tables

**Table 1:** Demographics and clinical characteristics of pediatric OSA patients undergoing DISE after previous adenoidectomy.

Variable	*N*	%
Gender		
Male	35	62.5
Female	21	37.5
Age (mean ± 95% CI, years)		
Initial adenoidectomy	5.36 ± 0.91	-
First DISE	7.11 ± 0.93	-
Timeframe (mean ± 95% CI, years)		
Time between initial adenoidectomy and DISE	1.75 ± 0.39	-
Time between adenoidectomies	2.37 ± 0.45	-
Mean AHI (mean ± 95% CI, events/hour)		
Pre-DISE	8.53 ± 4.5	
Improvement after DISE-related surgery	−6.33 ± 5.9	
BMI for age percentile at first DISE		
Group 1 underweight (<25%)	7	12.5
Group 2 normal weight (25% to <85%)	18	32.1
Group 3 overweight (85% to <95%)	5	8.9
Group 4 obese (95%+)	26	46.4
Comorbid conditions at time of first DISE		
Allergic rhinitis	26	46.4
Asthma	20	35.7
Developmental delay^†^	16	28.6
Reflux	18	32.1
Behavioral disorders	18	32.1
Hypertension	5	8.9
Laryngomalacia	6	10.7
Follow-up sleep surgery after DISE	48	85.7

OSA, obstructive sleep apnea; DISE, drug induced sleep endoscopy; CI, confidence interval; AHI, apnea-hypopnea index; BMI, body mass index. ^†^Secondary to autism, hypotonia, muscular dystrophy, trisomy 21, trisomy 12, fragile X, and etiology unknown.

**Table 2:** Adenoid characteristics.

Variable	*N*	%
Initial adenoidectomy technique		
Suction electrocautery	47	83.9
Coblation	9	16.1
Initial surgery		
Tonsillectomy and adenoidectomy	47	83.9
Adenoidectomy	9	16.1
Preoperative adenoid size (mean ± 95% CI)	2.55 ± 0.24	
Grade 0; 0%	0	0
Grade 1; <40%	7	13.5
Grade 2: 41%–70%	21	40.4
Grade 3; 71%–90%	18	34.6
Grade 4; 91%–100%	10	19.2
Postoperative adenoid size by DISE (mean ± 95% CI)	1.64 ± 0.33	
Grade 0; 0%	11	19.6
Grade 1; <40%	20	35.7
Grade 2; 41%–70%	9	16.1
Grade 3; 71%–90%	10	17.9
Grade 4; 91%–100%	6	10.7
Regrowth as determined by DISE	25	44.6
# undergoing revision adenoidectomy	32	57.1

DISE, drug induced sleep endoscopy; CI, confidence interval.

**Table 3:** Factors associated with adenoid regrowth as determined by DISE.

Variable	Regrowth	Absent/mild adenoid	*P* value
Males/females, %	60.0/40.0	64.5/34.5	0.73
Diagnosis of allergic rhinitis, %	56.0	38.7	0.20
Diagnosis of asthma, %	40.0	32.3	0.55
Diagnosis of behavioral issues, %	20.0	41.9	0.08
Diagnosis of developmental delay, %	20.0	35.5	0.20
Diagnosis of reflux, %	32.0	32.3	0.98
Initial Adenoid Size, Grades 0–4 (mean ± 95% CI)	2.77 ± 0.42	2.23 ± 0.33	^†^0.01
BMI percentile, groups 1–4 per Table 1 (mean ± 95% CI)	3.28 ± 0.39	2.56 ± 0.42	^†^0.02
Prior surgery adenoidectomy only, %	8.0	22.6	0.14
coblation technique, %	8.0	22.6	0.14
Age at adenoidectomy, mean ± 95% CI	5.12 ± 1.04	5.55 ± 1.52	0.31
Age at DISE, mean ± 95% CI	7.36 ± 1.11	6.91 ± 1.56	0.19
Time between adenoidectomy & DISE, mean ± 95% CI	2.24 ± 0.59	1.36 ± 0.52	^*∗*^0.05

DISE, drug induced sleep endoscopy; CI, confidence interval; BMI, body mass index. ^†^Significance with Kruskal-Wallis test. ^*∗*^Significance with independent samples *T*-testing.
